# Ultrasound to the Rescue: Axillary Clearance under Complete Regional Blockade

**DOI:** 10.1155/2021/6655930

**Published:** 2021-02-11

**Authors:** B. M Munasinghe, N. Subramaniam, S. Nimalan, P. Sivamayuran

**Affiliations:** District General Hospital, Mannar, Sri Lanka

## Abstract

No single regional anaesthetic technique is capable of complete anaesthesia of the axillary region. Regional or interfascial nerve blockade could be an effective alternative where administering general anaesthesia is not feasible, with superior analgesia, favourable haemodynamics, and reduced opiate related adverse effects. Ultrasound guidance improves effectiveness and safety profile. We report a case of a successful axillary clearance conducted under combined regional blocks for an axillary nodal recurrence following mastectomy for a breast carcinoma, in a patient who was not fit for general anaesthesia due to a persistent lobar pneumonia and recurrent asthma exacerbations. Our experience and current evidence supersede the initial conceptions of difficult ultrasonic intercostobrachial nerve (ICBN) visualization.

## 1. Introduction

Both the brachial plexus and the lateral cutaneous branches of the upper intercostal nerves (Intercostobrachial nerve T 2-3) are involved in the sensory innervation of the axillary region [1]. Analgesia of the axilla is possible with the use of systemic analgesics or ultrasound-guided high-volume plexus, fascial plane blocks, or relatively low-volume targeted nerve blocks such as intercostobrachial nerve (ICBN) blocks [2]. The fascial plane blocks of interest for this particular anatomical region are several, including pectoralis 2 and serratus anterior plane blocks. In general, these are relatively nonselective. The presence of underlying fibrous tissue and the scar subsequent to the earlier mastectomy made these blocks impractical in our patient. On the contrary, ICBN was more feasible due to a more lateral entry of the block needle. Traditionally, ICBN block has been carried out by the blind, local infiltration method. Several relatively new ultrasound-guided techniques have made it possible, either to selectively or via aforementioned fascial plane blocks, to block this smaller peripheral nerve, associated with higher success rates than the former [3]. Overall, these forms of anaesthesia are beneficial for patients not fit for general anaesthesia, by avoidance of systemic analgesics and their general detrimental effects. This case report presents an uncomplicated, unilateral supraclavicular brachial plexus (SBP) and ICBN blockade performed under ultrasound guidance, for an axillary clearance for a recurrent breast carcinoma, on a patient deemed unsuitable for general anaesthesia.

## 2. Case Report

**Figure 1:**
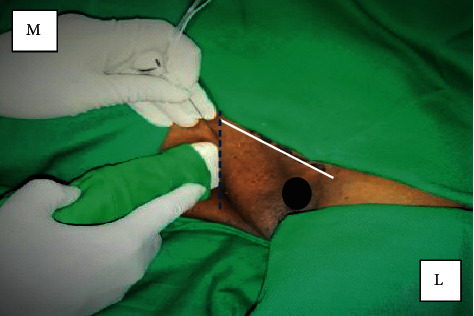
ICBN blockade using the linear probe using the in-plane approach. Black oval indicates the humeral head; white (solid line) indicates the anterior axillary fold, and blue (dotted) line indicates the 4th intercostal space. M: medial; L: lateral.

**Figure 2:**
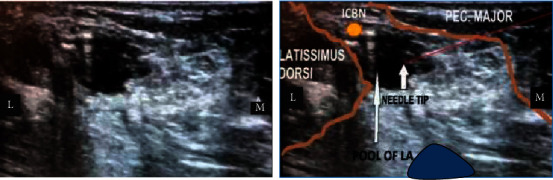
(a) The left panel shows ultrasonic view. (b) The right panel shows ultrasonic view with illustrations. Blue oval indicates the axillary vein. M: medial; L: lateral; ICBN: intercostobrachial nerve.

A 64-year-old patient presented with lymph node recurrence of a breast carcinoma in the left axillary region following an earlier left-sided mastectomy. She was a diagnosed patient with bronchial asthma with recurrent episodes of wheezing recently. At the time of presentation, she had right lower-zone pneumonia. She was seen by the medical team and started on broad-spectrum antibiotics. As the bronchial asthma has worsened with persistent pneumonia, pulmonology opinion was sought. Contrast-enhanced CT chest confirmed the right lower-lobe pneumonia and excluded any pulmonary metastasis. Recent history of chemotherapy (for the malignancy) suggested a relatively immunosuppressed state. Supportive therapy was provided in the form of high-protein diet and regular chest physiotherapy. Three weeks into the illness, ultrasound-guided  SBP and ICBN blocks were planned following a detailed discussion between the patient, surgeon, anaesthetist, and the pulmonologist. The patient was not septic clinically and maintained a peripheral saturation of 90% on air. There was no coagulopathy clinically or biochemically. Informed written consent was taken, and an intensive care bed was reserved. Regular nebulization was continued with the rest of medication. The next day, at the operating theatre, standard monitoring including peripheral saturation, noninvasive blood pressure, and ECG were established. Supplemental oxygen was provided via a face mask. Aseptic technique was adhered to. The linear ultrasound probe, 5–12 MHz (Samsung Medison Co., Ltd., Seoul, Korea), and a 22-gauge, 50 mm, single-use, Stimuplex^®^ (B. Braun Medical Inc., Melsungen, Germany) cannula were used for the nerve blocks. Initially, left-sided in-plane SBP block was performed ultrasonically using 20 ml of 0.25% plain bupivacaine, with 50 *µ*g of intravenous fentanyl as sedation. Subsequently, a left in-plane ICBN block was performed. The left hand was abducted; the probe was placed at the 4th intercostal space at the mid-axillary line parallel to the space. The needle was guided from medial to lateral (Figure 1), following the identification of the ICBN (Figure 2) at a rough depth of 0.8 cm. Ten milliliters of 0.25% plain bupivacaine was administered surrounding the ICBN.

Repetitive aspiration and real-time visualization with low-pressure injections were adequate to exclude intravenous and intraneuronal injections, respectively, in both nerve blocks (nerve stimulator was not available for the latter). Vital parameters were continuously monitored. Sensory blockade was assessed at 5-minute intervals by checking cold sensation around the axillary region. Satisfactory analgesia was achieved by 20 minutes. Surgery was conducted without the need of additional analgesia. Following a 45-minute surgery, she was sent to the intensive care unit for monitoring and supplemental oxygen therapy. The patient had satisfactory analgesia up to 12 hours, after which there was no residual nerve blockade, and she was discharged to the ward the next day.

## 3. Discussion

Axilla is a pyramidal-shaped area bounded by axillary folds which are primarily formed by groups of muscles. Sensory innervation of axilla is mainly by ICBN, which is responsible for the major portion of cutaneous supply. The brachial plexus also contributes to the sensory supply by innervating structures contained within the axilla, blockade of which could be required during extensive surgeries of the axilla, as a supplement to the former.

The ICBN is frequently given off as a cutaneous branch of the second intercostal nerve. It traverses the serratus anterior and crosses the axilla towards the medial side of the arm. It is anatomically separately located in relation to brachial plexus and thus not blocked by brachial plexus blocks [4]. However, ICBN can be blocked using several techniques. The traditional procedure is a subcutaneous ring infiltration on the dorsomedial upper arm, spanning the entire width of the medial aspect of the arm at the level of the axilla, usually from anterior to posterior direction [5]. This blind method has been associated with higher failure rates [5, 6]. Thoracic paravertebral, Pecs II, and serratus anterior blocks are other alternative methods of ICBN block. As ICBN is a relatively smaller and superficial nerve which could be difficult to visualize ultrasonically, selective blockade was believed to be difficult. However, several studies have provided convincing evidence to refute this [3, 6], favouring ultrasound guidance with superior analgesia and lesser adverse effects. A case series on management of intercostobrachial neuralgia in breast cancer patients by ICBN block suggests the safety of ultrasound guidance due to the presence of vascular structures around the ICBN and that the “injectate can be used to dissect tissue from around the nerve, potentially reducing any scar tissue compression of the nerve” [7].

Pecs II and serratus anterior fascial plane blocks are relatively high volume and nonselective. These are performed at the level of the 4th and 5th intercostal spaces (with slight variations according to patients) in anterior and just lateral to anterior axillary line, respectively. General anaesthesia was not feasible for this patient. The surgical scar and fibrous tissue made the former regional techniques impractical. Thus, selective ICBN block with supraclavicular brachial plexus were chosen for this patient. Ipsilateral diaphragmatic paralysis was a concern following the brachial plexus block due to the possibility of phrenic nerve blockade [8]; therefore, continuous monitoring for deterioration of respiratory parameters was carried out.

**Figure 3:**
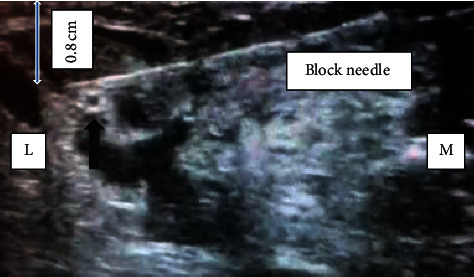
ICBN with block needle positioned immediately before local anaesthetic infiltration. Black arrow indicates the hyperechoic ICBN (M: medial; L: lateral).

The ICBN block was performed as follows. The patient was positioned supine with the left upper limb abducted to 90°. The ultrasound probe was placed at the apex of the axilla and humeral head, and axillary artery and vein were visualized initially in short-axis view at a depth of 2.5 cm. The probe was moved caudally until the axillary vein moved deeper in the posteromedial aspect of the pectoralis major. The ICBN was visualized at a rough depth of 0.8 cm appearing as a noncompressible oval, hyperechoic structure. Injection of 0.25% plain bupivacaine 10 ml was done surrounding the nerve (Figure 3).

Some ultrasound-based studies have suggested even lesser volumes [3]. Given the relative inexperience on ultrasound-guided ICBN blockade, the authors opted for a higher volume to avoid failure of the block. Real-time use of colour mode and repetitive aspiration with low-pressure injections excluded intravascular and intraneuronal entry (in the absence of nerve stimulator). The patient did not require additional analgesia throughout the procedure. Supplemental oxygen requirement was static.

## 4. Conclusion

Isolated ICBN block could be safely and effectively performed with the use of ultrasound. This could be specially useful for frail patients not suitable for general anaesthesia or other fascial planes of ICBN block, having potential benefits of avoidance of general effects of systemic anaesthetics and analgesics such as opiates. In the not far distant future, relatively easier, minimal volume, safer nerve block techniques would make ultrasound-guided nerve blocks far superior and ubiquitous.

## Data Availability

In addition to patient-related data and related references, no other data were utilized for the compilation of this paper.
